# Supplementary material for: Effects of different light intensities on lettuce growth, yield, and energy consumption optimization under uniform lighting conditions
Source: PeerJ. 2025 Mar 31;13:e19229. doi: 10.7717/peerj.19229 (PMC11967408; doi:10.7717/peerj.19229)
Supplement: Supplemental Information 1 [file peerj-13-19229-s001.docx]

**CONTENTS**

**[Fig4 PPFD Distribution of the Supplementary Lighting Plane in the P225 1](#_Toc8516)**

**[Fig5 The influence of different light intensities on the leaf amplitude, leaf width, and leaf length of Butterhead lettuce (ACE) and Spanish green lettuce (BDF) under the same light quality ratio.Mean values with standard error of mean (n = 4). 2](#_Toc18796)**

**[Butterhead lettuceleaf amplitude (Fig5A) 2](#_Toc12173)**

**[Butterhead lettuceleaf width (Fig5C) 2](#_Toc14793)**

**[Butterhead lettuce leaf length (Fig5E) 3](#_Toc7149)**

**[Spanish green lettuceleaf amplitude (Fig5B) 3](#_Toc3822)**

**[Spanish green lettuce leaf width (Fig5D) 3](#_Toc21970)**

**[Spanish green lettuce leaf length (Fig5F) 4](#_Toc7389)**

**[Fig6 The influence of different light intensities on the weight and dry matter content of Butterhead lettuce (AC) and Spanish green lettuce (BD) under the same light quality ratio 5](#_Toc16966)**

**[Fresh weight distribution of each Spanish green lettuce(Fig 6A) 5](#_Toc14688)**

**[Fresh weight distribution of each Butterhead lettuce(Fig 6B) 6](#_Toc22850)**

**[Dry matter content of each lettuce(Fig 6C,D) 7](#_Toc30843)**

**[Fig7 Changes in production efficiency ratio of Butterhead lettuce (A) and Spanish green lettuce (B) 9](#_Toc13031)**

**[Fig8 The effect of different light intensities on the SPAD values of Butterhead lettuce (A) and Spanish green lettuce (B) 10](#_Toc15692)**

**[Fig9 Photosynthetic performance of Butterhead lettuce (ACE) and Spanish green lettuce (BDF) under different light intensities. The box represents the Interquartile Range (IQR), the horizontal line within the box indicates the median value, the black dot on the box signifies the mean value, the whiskers at both ends of the box are used to distinguish outliers, and the diamond-shaped dots outside the box represent the outliers. 11](#_Toc11090)**

**[Supplementary materials 1 14](#_Toc19215)**

# **Fig4 PPFD Distribution of the Supplementary Lighting Plane in the P225**

| **201** | **219** | **232** | **221** | **234** | **224** | **197** |
| --- | --- | --- | --- | --- | --- | --- |
| **206** | **234** | **235** | **242** | **238** | **237** | **207** |
| **209** | **239** | **240** | **247** | **241** | **235** | **203** |
| **198** | **243** | **243** | **247** | **243** | **239** | **199** |
| **190** | **228** | **233** | **234** | **235** | **227** | **189** |
| **193** | **218** | **231** | **235** | **232** | **216** | **182** |
| **201** | **231** | **241** | **245** | **245** | **227** | **193** |
| **202** | **230** | **238** | **243** | **240** | **225** | **183** |
| **198** | **200** | **209** | **209** | **206** | **189** | **180** |

# **Fig5 The influence of different light intensities on the leaf amplitude, leaf width, and leaf length of Butterhead lettuce (ACE) and Spanish green lettuce (BDF) under the same light quality ratio.Mean values with standard error of mean (n = 4).**

## **Butterhead lettuceleaf amplitude (Fig5A)**

|  | **PPFD:125** | **yEr±** | **PPFD:150** | **yEr±** | **PPFD:175** | **yEr±** | **PPFD:200** | **yEr±** |
| --- | --- | --- | --- | --- | --- | --- | --- | --- |
| **20d** | **9.23** | **0.7** | **7.87** | **0.75** | **8.48** | **0.56** | **8.33** | **0.13** |
| **25d** | **18.03** | **0.8** | **17.65** | **0.3** | **17.73** | **1.46** | **16.8** | **0.61** |
| **30d** | **20.72** | **0.55** | **20.17** | **0.51** | **19.23** | **2.35** | **18.87** | **0.73** |
| **35d** | **21** | **0.25** | **20.95** | **0.4** | **21.18** | **0.55** | **21.02** | **0.69** |
|  | **PPFD:225** | **yEr±** | **PPFD:250** | **yEr±** | **PPFD:275** | **yEr±** | **PPFD:300** | **yEr±** |
| **20d** | **8.35** | **0.4** | **7** | **0.8** | **8.1** | **0.44** | **8.32** | **0.51** |
| **25d** | **18.3** | **0.3** | **17.05** | **0.83** | **18.03** | **0.87** | **19.18** | **0.55** |
| **30d** | **20.42** | **0.52** | **20.35** | **0.44** | **19.82** | **0.79** | **20.12** | **0.44** |
| **35d** | **22.25** | **0.26** | **20.97** | **0.75** | **20.87** | **0.64** | **20.92** | **0.38** |

## **Butterhead lettuceleaf width (Fig5C)**

|  | **PPFD:125** | **yEr±** | **PPFD:150** | **yEr±** | **PPFD:175** | **yEr±** | **PPFD:200** | **yEr±** |
| --- | --- | --- | --- | --- | --- | --- | --- | --- |
| **20d** | **3.87** | **0.42** | **3.97** | **0.49** | **3.83** | **0.21** | **4.13** | **0.7** |
| **25d** | **5.6** | **0.52** | **5.57** | **0.55** | **5.27** | **0.67** | **5.6** | **0.1** |
| **30d** | **6.7** | **0.3** | **6.77** | **0.21** | **7.5** | **0.46** | **6.97** | **0.55** |
| **35d** | **7.2** | **0.26** | **7.63** | **0.45** | **8.47** | **0.85** | **8.5** | **0.61** |
| **40d** | **9** | **0.5** | **8.2** | **0.1** | **8.9** | **0.25** | **8.7** | **0.5** |
|  | **PPFD:225** | **yEr±** | **PPFD:250** | **yEr±** | **PPFD:275** | **yEr±** | **PPFD:300** | **yEr±** |
| **20d** | **4.37** | **0.21** | **4.23** | **0.32** | **4.4** | **0.44** | **4.33** | **0.5** |
| **25d** | **6** | **0.35** | **6** | **0.2** | **6** | **0.36** | **6.5** | **0.2** |
| **30d** | **7.53** | **0.81** | **7.33** | **0.31** | **7.17** | **0.76** | **7.83** | **0.58** |
| **35d** | **8.77** | **0.4** | **8.37** | **0.42** | **8.5** | **0.95** | **9.17** | **0.76** |
| **40d** | **9.27** | **0.38** | **9.13** | **0.15** | **9.23** | **0.06** | **9.83** | **0.61** |

## **Butterhead lettuce leaf length (Fig5E)**

|  | **PPFD:125** | **yEr±** | **PPFD:150** | **yEr±** | **PPFD:175** | **yEr±** | **PPFD:200** | **yEr±** |
| --- | --- | --- | --- | --- | --- | --- | --- | --- |
| **20d** | **6.3** | **0.1** | **6.6** | **0.62** | **7.47** | **1.12** | **6.63** | **0.32** |
| **25d** | **9.07** | **0.32** | **8.6** | **0.35** | **9.03** | **0.84** | **8.67** | **0.25** |
| **30d** | **10.23** | **1.08** | **9.57** | **0.51** | **10.27** | **1.21** | **10.2** | **0.52** |
| **35d** | **11.23** | **0.8** | **11** | **0.1** | **11.3** | **0.35** | **11.86** | **0.32** |
| **40d** | **13.8** | **0.62** | **13.17** | **0.12** | **13.5** | **0.55** | **13.17** | **0.74** |
|  | **PPFD:** | **yEr±** | **PPFD:250** | **yEr±** | **PPFD:275** | **yEr±** | **PPFD:300** | **yEr±** |
| **20d** | **6.37** | **0.25** | **6.37** | **0.78** | **6.63** | **0.46** | **6.67** | **0.61** |
| **25d** | **9** | **0.87** | **8.4** | **1.28** | **9.23** | **0.55** | **8.87** | **0.25** |
| **30d** | **10.53** | **0.5** | **10** | **0.95** | **9.63** | **0.55** | **10.77** | **1.12** |
| **35d** | **11.3** | **0.72** | **11.77** | **0.59** | **11** | **0.95** | **11.33** | **1.04** |
| **40d** | **14.03** | **0.65** | **12.23** | **0.95** | **12.9** | **0.52** | **12.6** | **0.95** |

## **Spanish green lettuce leaf amplitude (Fig5B)**

|  | **PPFD:125** | **yEr±** | **PPFD:150** | **yEr±** | **PPFD:175** | **yEr±** | **PPFD:200** | **yEr±** |
| --- | --- | --- | --- | --- | --- | --- | --- | --- |
| **20d** | **9.75** | **0.43** | **8.98** | **0.81** | **9.55** | **0.09** | **9.2** | **0.4** |
| **25d** | **18.22** | **0.6** | **18.18** | **0.81** | **17.18** | **1.01** | **17.38** | **1.32** |
| **30d** | **21.5** | **0.87** | **20.27** | **0.87** | **20** | **0.25** | **19.83** | **0.58** |
| **35d** | **24.65** | **0.69** | **23.17** | **0.76** | **22.5** | **0.66** | **20.42** | **0.52** |
|  | **PPFD:225** | **yEr±** | **PPFD:250** | **yEr±** | **PPFD:275** | **yEr±** | **PPFD:300** | **yEr±** |
| **20d** | **8** | **0.5** | **7.88** | **0.33** | **8.87** | **1.51** | **8.58** | **0.42** |
| **25d** | **16.42** | **1.63** | **15.28** | **1.97** | **17.48** | **1.58** | **18.12** | **0.55** |
| **30d** | **18.08** | **1.32** | **17.1** | **0.72** | **18.9** | **0.22** | **18.56** | **1.18** |
| **35d** | **20.25** | **2.14** | **19.5** | **1.63** | **20.33** | **0.8** | **20.92** | **1.04** |

## **Spanish green lettuce leaf width (Fig5D)**

|  | **PPFD:125** | **yEr±** | **PPFD:150** | **yEr±** | **PPFD:175** | **yEr±** | **PPFD:200** | **yEr±** |
| --- | --- | --- | --- | --- | --- | --- | --- | --- |
| **20d** | **5.1** | **0.53** | **4.67** | **0.21** | **4.83** | **0.23** | **4.93** | **0.4** |
| **25d** | **6.97** | **0.42** | **6.73** | **0.21** | **6.7** | **0.53** | **7.53** | **0.7** |
| **30d** | **8.67** | **1.15** | **8.4** | **1.28** | **8.77** | **0.32** | **8.9** | **0.17** |
| **35d** | **9.93** | **0.4** | **9.8** | **0.35** | **9.9** | **0.56** | **9.83** | **0.58** |
| **40d** | **11.4** | **1.15** | **10.87** | **0.38** | **12** | **0.3** | **11.17** | **0.45** |
|  | **PPFD:225** | **yEr±** | **PPFD:250** | **yEr±** | **PPFD:275** | **yEr±** | **PPFD:300** | **yEr±** |
| **20d** | **5.37** | **0.21** | **5** | **0.26** | **5.9** | **0.98** | **5.2** | **0.26** |
| **25d** | **7.6** | **0.66** | **6.87** | **0.57** | **7.87** | **0.4** | **7.47** | **0.35** |
| **30d** | **8.67** | **0.76** | **8.27** | **0.67** | **8.93** | **0.86** | **9.37** | **0.78** |
| **35d** | **9.9** | **0.66** | **10.5** | **0.26** | **10.47** | **0.4** | **10.23** | **1.62** |
| **40d** | **10.5** | **0.8** | **10.83** | **0.67** | **11.23** | **1.27** | **10.6** | **0.66** |

## **Spanish green lettuce leaf length (Fig5F)**

|  | **PPFD:125** | **yEr±** | **PPFD:150** | **yEr±** | **PPFD:175** | **yEr±** | **PPFD:200** | **yEr±** |
| --- | --- | --- | --- | --- | --- | --- | --- | --- |
| **20d** | **8.17** | **0.59** | **7.8** | **0.44** | **8.13** | **0.21** | **7.2** | **0.61** |
| **25d** | **10.63** | **0.12** | **10** | **0.35** | **9.8** | **0.82** | **9.63** | **0.57** |
| **30d** | **12.17** | **0.76** | **12.13** | **0.6** | **11.63** | **0.71** | **11.43** | **0.83** |
| **35d** | **13.53** | **0.06** | **13.87** | **0.55** | **12.27** | **0.15** | **12.33** | **0.76** |
| **40d** | **16** | **0.35** | **16.07** | **0.6** | **15.9** | **0.17** | **15.73** | **0.25** |
|  | **PPFD:125** | **yEr±** | **PPFD:150** | **yEr±** | **PPFD:175** | **yEr±** | **PPFD:200** | **yEr±** |
| **20d** | **7.37** | **0.38** | **7** | **0.35** | **7.63** | **0.15** | **7.37** | **0.57** |
| **25d** | **9.83** | **0.67** | **8.73** | **0.71** | **9.87** | **0.32** | **10** | **0.46** |
| **30d** | **10.07** | **0.4** | **9.2** | **0.26** | **11.47** | **0.72** | **10.47** | **0.15** |
| **35d** | **12.23** | **2.03** | **12.1** | **0.46** | **13.17** | **0.42** | **12.2** | **0.72** |
| **40d** | **14** | **1.45** | **13.7** | **0.44** | **15** | **1.8** | **14.33** | **1.26** |

# **Fig6 The influence of different light intensities on the weight and dry matter content of Butterhead lettuce (AC) and Spanish green lettuce (BD) under the same light quality ratio**

## **Fresh weight distribution of each Spanish green lettuce(Fig 6A)**

| **PPFD:125** | **a** | **36** | **42** | **35** | **42** | **41** | **37** | **36** |
| --- | --- | --- | --- | --- | --- | --- | --- | --- |
|  | **b** | **29** | **43** | **45** | **42** | **43** | **36** | **30** |
|  | **c** | **21** | **32** | **33** | **55** | **43** | **27** | **29** |
|  | **d** | **30** | **29** | **31** | **33** | **34** | **32** | **25** |
|  | **e** | **23** | **34** | **30** | **28** | **39** | **29** | **24** |
|  | **f** | **18** | **32** | **29** |  | **33** | **30** | **25** |
| **PPFD:150** | **a** | **30** | **47** | **37** | **38** | **38** | **38** | **37** |
|  | **b** | **31** | **47** | **47** | **42** | **41** | **42** | **30** |
|  | **c** | **36** | **49** | **46** | **42** | **37** | **40** | **36** |
|  | **d** | **19** | **38** | **43** | **41** | **41** | **42** | **35** |
|  | **e** | **32** | **33** | **40** | **30** | **47** | **41** | **30** |
|  | **f** | **24** | **34** | **44** | **36** | **33** | **46** | **29** |
| **PPFD:175** | **a** | **37** | **43** | **42** | **48** | **33** | **42** | **40** |
|  | **b** | **35** | **46** | **47** | **43** | **50** | **49** | **37** |
|  | **c** | **40** | **49** | **55** | **57** | **50** | **46** | **37** |
|  | **d** | **39** | **48** | **49** | **54** | **46** | **42** | **34** |
|  | **e** | **37** | **39** | **36** | **39** | **41** | **34** | **33** |
|  | **f** | **26** | **35** |  | **38** | **43** | **39** | **20** |
| **PPFD:200** | **a** | **45** | **51** | **62** | **50** | **66** | **60** | **40** |
|  | **b** | **41** | **53** | **57** | **58** | **49** | **54** | **40** |
|  | **c** | **47** | **50** | **49** | **56** | **53** | **53** | **30** |
|  | **d** | **34** | **42** | **57** | **44** | **55** | **51** | **38** |
|  | **e** | **46** | **43** | **53** | **50** | **52** | **51** | **37** |
|  | **f** | **36** | **42** | **52** | **54** | **60** | **48** | **34** |
| **PPFD:225** | **a** | **34** | **47** | **45** | **53** | **50** | **36** | **42** |
|  | **b** | **32** | **61** | **44** | **45** | **47** | **58** | **30** |
|  | **c** | **36** | **46** | **54** | **69** | **47** | **49** | **29** |
|  | **d** | **37** | **32** | **52** | **39** | **58** | **53** | **29** |
|  | **e** | **26** | **37** | **50** | **37** | **38** | **40** | **41** |
|  | **f** | **36** | **40** | **35** |  | **48** | **38** | **39** |
| **PPFD:250** | **a** | **38** | **47** | **38** | **62** | **54** | **42** | **52** |
|  | **b** | **34** | **64** | **46** | **49** | **45** | **49** | **30** |
|  | **c** | **37** | **48** | **60** | **43** | **46** | **50** | **42** |
|  | **d** | **47** | **54** | **46** | **63** | **52** | **52** | **47** |
|  | **e** | **45** | **56** | **57** | **50** | **55** | **44** | **35** |
|  | **f** | **36** | **43** | **45** | **40** | **36** | **51** | **45** |
| **PPFD:275** | **a** | **36** | **56** | **31** | **45** | **70** | **57** | **49** |
|  | **b** | **35** | **68** | **62** | **70** | **56** | **49** | **45** |
|  | **c** | **32** | **39** | **57** | **64** | **61** | **50** | **34** |
|  | **d** | **26** | **45** | **74** | **80** | **40** | **70** | **58** |
|  | **e** | **28** | **55** | **55** | **71** | **71** | **58** | **40** |
|  | **f** | **41** | **62** | **47** |  | **52** | **58** | **40** |
| **PPFD:300** | **a** | **46** | **62** | **53** | **61** | **58** | **71** | **52** |
|  | **b** | **52** | **71** | **68** | **67** | **68** | **62** | **49** |
|  | **c** | **45** | **48** | **80** | **68** | **64** | **65** | **49** |
|  | **d** | **39** | **73** | **62** | **69** | **73** | **62** | **52** |
|  | **e** | **52** | **75** | **63** | **81** | **68** | **43** | **42** |
|  | **f** | **45** | **63** | **78** | **36** | **61** | **69** | **28** |

## **Fresh weight distribution of each Butterhead lettuce(Fig 6B)**

| **PPFD:125** | **a** | **24** | **27** | **39** | **33** | **27** | **34** | **33** |
| --- | --- | --- | --- | --- | --- | --- | --- | --- |
|  | **b** | **28** | **40** | **46** | **49** | **34** | **34** | **24** |
|  | **c** | **24** | **36** | **31** | **35** | **36** | **37** | **24** |
|  | **d** | **25** | **36** | **39** | **22** | **39** | **29** | **23** |
|  | **e** | **29** | **37** | **35** | **39** | **43** | **39** | **26** |
|  | **f** | **59** | **33** | **36** | **33** | **37** | **41** | **23** |
| **PPFD:150** | **a** | **27** | **38** | **45** |  | **47** | **38** | **23** |
|  | **b** | **26** | **35** | **42** | **42** | **41** | **27** | **29** |
|  | **c** | **39** | **39** | **38** | **50** | **38** | **40** | **24** |
|  | **d** | **32** | **46** | **47** | **41** | **45** | **33** | **34** |
|  | **e** | **29** | **42** | **55** | **42** | **36** | **49** | **31** |
|  | **f** | **25** | **41** | **53** | **42** | **49** | **37** | **26** |
| **PPFD:175** | **a** | **29** | **46** | **47** | **55** | **45** | **44** | **29** |
|  | **b** | **41** | **52** | **49** | **55** | **46** | **47** | **35** |
|  | **c** | **47** | **47** | **52** | **56** | **64** | **54** | **30** |
|  | **d** | **40** | **51** | **63** | **51** | **44** | **45** | **45** |
|  | **e** | **37** | **56** | **52** | **54** | **51** | **47** | **44** |
|  | **f** | **41** | **40** | **48** | **53** | **46** | **38** | **36** |
| **PPFD:200** | **a** | **35** | **52** | **47** | **50** | **48** | **47** | **42** |
|  | **b** | **44** | **43** | **54** | **60** | **55** | **55** | **31** |
|  | **c** | **31** | **37** | **45** | **49** | **48** | **40** | **32** |
|  | **d** | **33** | **45** | **44** | **47** | **39** | **48** | **39** |
|  | **e** | **42** | **42** | **52** | **44** | **44** | **54** | **42** |
|  | **f** | **37** | **42** | **39** | **56** | **59** | **56** | **24** |
| **PPFD:225** | **a** | **45** | **31** | **58** | **55** | **54** | **36** | **28** |
|  | **b** | **39** | **42** | **60** | **42** | **45** | **52** | **30** |
|  | **c** | **34** | **45** | **49** | **41** | **53** | **33** | **54** |
|  | **d** | **52** | **62** | **47** | **58** | **60** | **60** | **52** |
|  | **e** | **42** | **50** | **46** | **65** | **47** | **49** | **30** |
|  | **f** | **36** | **54** | **41** | **63** | **54** | **63** | **48** |
| **PPFD:250** | **a** | **42** | **46** | **56** |  | **54** | **52** | **45** |
|  | **b** | **38** | **35** | **51** | **59** | **59** | **52** | **38** |
|  | **c** | **53** | **52** | **56** | **54** | **55** | **42** | **56** |
|  | **d** | **56** | **56** | **56** | **62** | **60** | **50** | **42** |
|  | **e** | **36** | **60** | **49** | **56** | **45** | **53** | **39** |
|  | **f** | **46** | **64** | **61** | **61** | **55** | **56** | **54** |
| **PPFD:275** | **a** | **29** | **56** | **52** | **65** | **51** | **36** | **28** |
|  | **b** | **45** | **65** | **44** | **48** | **41** | **47** | **62** |
|  | **c** | **38** | **52** | **65** | **34** | **63** | **45** | **38** |
|  | **d** | **53** | **62** | **56** | **59** | **57** | **47** | **44** |
|  | **e** | **49** | **52** | **65** | **57** | **56** | **52** | **49** |
|  | **f** | **37** | **68** | **72** | **54** | **52** | **49** | **41** |
| **PPFD:300** | **a** | **57** | **67** | **50** | **73** | **53** | **51** | **37** |
|  | **b** | **63** | **66** | **76** | **74** | **66** | **53** | **68** |
|  | **c** | **51** | **70** | **76** | **68** | **59** | **55** | **55** |
|  | **d** | **54** | **48** | **52** | **58** | **62** | **42** | **54** |
|  | **e** | **63** | **70** | **75** | **68** | **62** | **67** | **53** |
|  | **f** | **66** | **66** | **66** | **70** | **74** | **77** | **38** |

## **Dry matter content of each lettuce(Fig 6C,D)**

| **Spanish green lettuce** | | | |
| --- | --- | --- | --- |
| **Group** | **DW(g)** | **FW(g)** | **Dry matter content(%)** |
| **PPFD:125** | **5.481** | **100** | **5.4800** |
| **PPFD:150** | **6.049** | **126** | **4.8000** |
| **PPFD:175** | **7.772** | **157** | **4.9500** |
| **PPFD:200** | **7.981** | **159** | **5.0200** |
| **PPFD:225** | **8.537** | **162** | **5.2700** |
| **PPFD:250** | **9.122** | **173** | **5.2700** |
| **PPFD:275** | **10.738** | **176** | **6.1000** |
| **PPFD:300** | **9.752** | **190** | **5.1300** |

| **Butterhead lettuce** | | | |
| --- | --- | --- | --- |
| **Group** | **DW(g)** | **FW(g)** | **Dry matter content(%)** |
| **PPFD:125** | **6.116** | **123** | **4.9700** |
| **PPFD:150** | **6.137** | **119** | **5.1600** |
| **PPFD:175** | **7.579** | **147** | **5.1600** |
| **PPFD:200** | **8.201** | **140** | **5.8600** |
| **PPFD:225** | **10.102** | **183** | **5.5200** |
| **PPFD:250** | **10.954** | **178** | **6.1500** |
| **PPFD:275** | **11.172** | **196** | **5.7000** |
| **PPFD:300** | **12.741** | **219** | **5.8200** |

# **Fig7 Changes in production efficiency ratio of Butterhead lettuce (A) and Spanish green lettuce (B)**

| **Group** | **Partial plant weight(g)** | | **Average weight(g)** | | **Power consumption(kw.h)** |
| --- | --- | --- | --- | --- | --- |
|  | **Butterhead lettuce** | **Spanish green lettuce** | **Butterhead lettuce** | **Spanish green lettuce** |  |
| **PPFD:125** | **123** | **100** | **36.64** | **36.6** | **2.47** |
| **PPFD:150** | **119** | **126** | **42** | **41.08** | **2.87** |
| **PPFD:175** | **147** | **157** | **50.44** | **45.12** | **3.27** |
| **PPFD:200** | **140** | **159** | **47.88** | **52.76** | **3.6** |
| **PPFD:225** | **183** | **162** | **51.24** | **47.48** | **4.11** |
| **PPFD:250** | **178** | **173** | **54.36** | **50.88** | **4.54** |
| **PPFD:275** | **196** | **176** | **54.48** | **58.16** | **4.87** |
| **PPFD:300** | **219** | **190** | **64.8** | **65.4** | **5.22** |

# **Fig8 The effect of different light intensities on the SPAD values of Butterhead lettuce (A) and Spanish green lettuce (B)**

| **Group** | **Test plants** | **Butterhead lettuce** | **Spanish green lettuce** |
| --- | --- | --- | --- |
| **PPFD:125** | **1** | **37.78** | **26.97** |
| **PPFD:125** | **2** | **36.28** | **29.47** |
| **PPFD:125** | **3** | **37.35** | **27.5** |
| **PPFD:125** | **4** | **37.14** | **27.98** |
| **PPFD:150** | **1** | **36.13** | **30.87** |
| **PPFD:150** | **2** | **36.9** | **30.47** |
| **PPFD:150** | **3** | **36.28** | **29.2** |
| **PPFD:150** | **4** | **36.44** | **30.18** |
| **PPFD:175** | **1** | **37.45** | **33.5** |
| **PPFD:175** | **2** | **38.4** | **33.27** |
| **PPFD:175** | **3** | **37.25** | **33.33** |
| **PPFD:175** | **4** | **37.7** | **33.37** |
| **PPFD:200** | **1** | **39.75** | **34.47** |
| **PPFD:200** | **2** | **40.05** | **34.77** |
| **PPFD:200** | **3** | **39.4** | **34.63** |
| **PPFD:200** | **4** | **39.73** | **34.62** |
| **PPFD:225** | **1** | **38.33** | **36.03** |
| **PPFD:225** | **2** | **38.8** | **36.8** |
| **PPFD:225** | **3** | **39.2** | **33.73** |
| **PPFD:225** | **4** | **38.78** | **35.52** |
| **PPFD:250** | **1** | **38.93** | **33.77** |
| **PPFD:250** | **2** | **37.23** | **32.17** |
| **PPFD:250** | **3** | **39.05** | **34.63** |
| **PPFD:250** | **4** | **38.4** | **33.52** |
| **PPFD:275** | **1** | **40.83** | **34.8** |
| **PPFD:275** | **2** | **40.63** | **35.03** |
| **PPFD:275** | **3** | **40.45** | **35.87** |
| **PPFD:275** | **4** | **40.64** | **35.23** |
| **PPFD:300** | **1** | **41.15** | **35.37** |
| **PPFD:300** | **2** | **40.65** | **34.7** |
| **PPFD:300** | **3** | **41.53** | **32.53** |
| **PPFD:300** | **4** | **41.11** | **34.2** |

# **Fig9 Photosynthetic performance of Butterhead lettuce (ACE) and Spanish green lettuce (BDF) under different light intensities. The box represents the Interquartile Range (IQR), the horizontal line within the box indicates the median value, the black dot on the box signifies the mean value, the whiskers at both ends of the box are used to distinguish outliers, and the diamond-shaped dots outside the box represent the outliers.**

| **Spanish green lettuce** | **PPFD: 125** | **PPFD: 150** | **PPFD: 175** | **PPFD: 200** | **PPFD: 225** | **PPFD: 250** | **PPFD: 275** | **PPFD: 300** |
| --- | --- | --- | --- | --- | --- | --- | --- | --- |
| **Tr** | **18.26** | **19.65** | **17.49** | **19.97** | **19.68** | **18.38** | **19.08** | **23.09** |
|  | **17.92** | **19.44** | **17.59** | **18.34** | **17.89** | **18.41** | **22.93** | **21.82** |
|  | **17.93** | **18.76** | **19.12** | **19.21** | **19.03** | **18.81** | **21.64** | **22.54** |
|  | **14.72** | **18.57** | **19** | **19.92** | **16.06** | **17.54** | **20.53** | **20.66** |
|  | **14.94** | **17.94** | **18.36** | **17.54** | **18.22** | **17.93** | **18.34** | **19.85** |
|  | **15.43** | **18.05** | **16.29** | **18.32** | **18.19** | **17.64** | **19.88** | **20.07** |
|  | **18.85** | **14.73** | **16.29** | **19.04** | **17.49** | **19.37** | **21.01** | **19.5** |
|  | **18.06** | **15.33** | **18.67** | **16.04** | **19.28** | **21.53** | **21.27** | **19.48** |
|  | **19.08** | **16.51** | **19.89** | **16.27** | **20.27** | **20.71** | **18.14** | **18.49** |
| **Pn** | **7.17** | **7.49** | **7.78** | **7.33** | **8.33** | **9.22** | **7.93** | **10.28** |
|  | **7.37** | **7.52** | **8.08** | **7.96** | **8.26** | **9** | **11.26** | **10.53** |
|  | **8.27** | **7.36** | **7.81** | **7.89** | **10.68** | **8.34** | **10.2** | **9.61** |
|  | **7.28** | **7.71** | **7.19** | **8.06** | **8.31** | **10.77** | **8.41** | **9.27** |
|  | **6.9** | **7.89** | **7.56** | **7.73** | **8.43** | **12.03** | **9** | **8.26** |
|  | **7.58** | **7.9** | **7.64** | **8.33** | **10.93** | **9.78** | **10.98** | **10.38** |
|  | **7.45** | **7.35** | **7.25** | **8.3** | **7.59** | **8.06** | **9** | **10.07** |
|  | **7.39** | **7.51** | **7.77** | **8.5** | **7.89** | **7.94** | **9.66** | **10.63** |
|  | **7.96** | **7.59** | **7.48** | **8.1** | **9.44** | **10.62** | **9.4** | **10.04** |
| **Gs** | **0.22** | **0.23** | **0.24** | **0.29** | **0.32** | **0.32** | **0.34** | **0.31** |
|  | **0.24** | **0.24** | **0.24** | **0.29** | **0.33** | **0.33** | **0.33** | **0.31** |
|  | **0.22** | **0.24** | **0.24** | **0.29** | **0.33** | **0.32** | **0.31** | **0.34** |
|  | **0.21** | **0.26** | **0.29** | **0.27** | **0.31** | **0.3** | **0.31** | **0.34** |
|  | **0.2** | **0.26** | **0.28** | **0.28** | **0.32** | **0.31** | **0.31** | **0.34** |
|  | **0.21** | **0.25** | **0.27** | **0.28** | **0.34** | **0.3** | **0.33** | **0.35** |
|  | **0.23** | **0.22** | **0.27** | **0.25** | **0.3** | **0.36** | **0.34** | **0.37** |
|  | **0.23** | **0.23** | **0.28** | **0.25** | **0.3** | **0.33** | **0.36** | **0.33** |
|  | **0.23** | **0.23** | **0.29** | **0.26** | **0.29** | **0.35** | **0.35** | **0.32** |

| **Butterhead lettuce** | **PPFD: 125** | **PPFD: 150** | **PPFD: 175** | **PPFD: 200** | **PPFD: 225** | **PPFD: 250** | **PPFD: 275** | **PPFD: 300** |
| --- | --- | --- | --- | --- | --- | --- | --- | --- |
| **Tr** | **10.42** | **7.47** | **6.42** | **11.77** | **10.29** | **12.3** | **16.75** | **12** |
|  | **11.48** | **7.58** | **6.99** | **12.15** | **10.9** | **12.53** | **13.08** | **11.3** |
|  | **10.68** | **7.79** | **7.84** | **12.92** | **12.3** | **11.03** | **16.77** | **11.68** |
|  | **9.8** | **8.57** | **10.77** | **11.34** | **12.42** | **11.64** | **11.21** | **13.53** |
|  | **8.93** | **8.35** | **10.9** | **7.95** | **12.85** | **12.42** | **11.78** | **14.63** |
|  | **9.16** | **8.25** | **10.75** | **9.31** | **12.08** | **12.63** | **13.04** | **15.12** |
|  | **7.03** | **11.19** | **10.4** | **12.38** | **13.24** | **11.16** | **11.9** | **9.61** |
|  | **7.14** | **11.39** | **10.67** | **12.4** | **15.24** | **11.27** | **8.05** | **9.88** |
|  | **8.49** | **10.94** | **11.23** | **7.54** | **12.74** | **11.45** | **9.02** | **10.23** |
| **Pn** | **8.461** | **8.508** | **8.753** | **12.395** | **9.319** | **9.452** | **11.29** | **10.452** |
|  | **8.73** | **8.354** | **8.522** | **10.239** | **11.3** | **9.73** | **10.885** | **9.671** |
|  | **8.394** | **8.487** | **8.718** | **9.675** | **10.172** | **10.074** | **10.298** | **9.115** |
|  | **9.231** | **9.7** | **8.768** | **8.773** | **10.167** | **10.336** | **11.608** | **14.056** |
|  | **10.093** | **8.378** | **9.34** | **9.653** | **10.64** | **10.154** | **10.231** | **11.103** |
|  | **8.623** | **9.507** | **9.073** | **10.111** | **9.946** | **12.024** | **9.869** | **11.557** |
|  | **8.994** | **9.404** | **9.393** | **9.338** | **10.395** | **10.093** | **9.426** | **10.372** |
|  | **8.648** | **8.702** | **9.163** | **8.854** | **10.324** | **10.365** | **10.438** | **9.719** |
|  | **8.588** | **10.175** | **9.742** | **9.181** | **10.097** | **10.365** | **9.312** | **9.731** |
| **Gs** | **0.28** | **0.32** | **0.24** | **0.32** | **0.34** | **0.33** | **0.3** | **0.36** |
|  | **0.28** | **0.32** | **0.25** | **0.32** | **0.3** | **0.32** | **0.34** | **0.33** |
|  | **0.28** | **0.29** | **0.27** | **0.29** | **0.27** | **0.33** | **0.35** | **0.41** |
|  | **0.3** | **0.27** | **0.29** | **0.31** | **0.32** | **0.32** | **0.37** | **0.32** |
|  | **0.29** | **0.26** | **0.3** | **0.32** | **0.31** | **0.34** | **0.4** | **0.38** |
|  | **0.28** | **0.27** | **0.3** | **0.3** | **0.31** | **0.34** | **0.37** | **0.41** |
|  | **0.25** | **0.29** | **0.33** | **0.27** | **0.34** | **0.32** | **0.35** | **0.31** |
|  | **0.24** | **0.29** | **0.32** | **0.27** | **0.35** | **0.32** | **0.33** | **0.32** |
|  | **0.23** | **0.28** | **0.31** | **0.29** | **0.34** | **0.33** | **0.32** | **0.37** |

# **Supplementary materials 1**

Data before adjusting the lighting fixtures:

| 110 | 175 | 182 | 171 | 163 | 174 | 175 | 167 | 101 |
| --- | --- | --- | --- | --- | --- | --- | --- | --- |
| 133 | 192 | 212 | 216 | 201 | 204 | 203 | 163 | 132 |
| 147 | 205 | 223 | 226 | 216 | 214 | 211 | 175 | 141 |
| 150 | 194 | 230 | 230 | 217 | 218 | 216 | 173 | 143 |
| 154 | 207 | 224 | 226 | 218 | 218 | 213 | 170 | 140 |
| 146 | 197 | 208 | 212 | 200 | 200 | 198 | 165 | 122 |
| 121 | 170 | 176 | 172 | 167 | 166 | 164 | 163 | 124 |

PPFD uniformity：55.8%
